# Effect of elexacaftor-tezacaftor-ivacaftor therapy on systemic inflammation in people with cystic fibrosis

**DOI:** 10.36416/1806-3756/e20250285

**Published:** 2026-03-05

**Authors:** Viviane Mauro Corrêa Meyer, Natália Aranha, Patricia Palmeira, Rodrigo Abensur Athanazio, Luiz Vicente Ribeiro Ferreira da Silva-Filho

**Affiliations:** 1. Instituto da Criança e do Adolescente, Hospital das Clínicas, Faculdade de Medicina, Universidade de São Paulo, São Paulo (SP) Brasil.; 2. Instituto do Coração - InCor - Hospital das Clínicas, Faculdade de Medicina, Universidade de São Paulo, São Paulo (SP) Brasil.

## TO THE EDITOR:

Cystic fibrosis (CF) is a multisystemic genetic disease caused by disturbances of expression or function of the CF transmembrane conductance regulator (CFTR) protein. In the airways, CFTR dysfunction results in hyperviscous mucus, chronic bacterial infection, and structural lung damage. Airway inflammation is multifactorial and a key component in CF pathophysiology. In addition to inflammation caused by bacterial colonization, CFTR defects lead to an imbalance in bioactive lipids and oxidative/antioxidative substances, as well as in mucin production. Recent studies indicate that several inflammatory cell types, including neutrophils, macrophages, and lymphocytes, may exhibit alterations in people with CF (pwCF). All of these factors contribute to an increased influx of neutrophils, to substantial release of proteases, and ultimately to lung remodeling.^1^ ^,^ ^2^

Recently, the introduction of highly effective CFTR modulator therapy with elexacaftor-tezacaftor-ivacaftor (ETI) has dramatically shifted the prognosis of CF. Pivotal studies have shown improved quality of life, body mass index, and lung function, along with a marked reduction in the number of exacerbation events.^3^ However, the impact of ETI on airway and systemic inflammation is still not fully understood. The current literature includes studies with small sample sizes that focus on only a few systemic biomarkers and often include patients who have previously used other modulators.^4^ ^-^ ^6^

This pilot study is an extension of a cohort study which recruited pwCF ≥ 6 years of age from the pediatric and adult CF clinics during a pulmonary exacerbation between September 2021 and March 2023. Follow up visits were performed at 1-3 months and 4-6 months, with lung function testing and measurement of blood inflammatory biomarkers at each visit. During this period, patients with at least one *F508del* mutation who were started on ETI per clinical protocol were enrolled in this ancillary study.

Data were collected from the last study visit before ETI initiation (T0) and at a subsequent monitoring visit (T1). We measured IL-1β, IL-2, IL-4, IL-6, IL-8, IL-10, IL-12, IL-17, TNF, and IFN-γ concentrations using a flow cytometer (FACS LRS II Fortessa; BD Biosciences, San Jose, CA, USA) and applying a cytokine assay kit (BD Cytometric Bead Array System; BD Biosciences). Chitinase 3-like-1 (also known as YKL-40), myeloperoxidase (MPO), matrix metalloproteinase 9, neutrophil elastase, and calprotectin were measured with ELISA kits (R&D Systems, Minneapolis, MN, USA). We hypothesized that ETI use would lead to a marked reduction in inflammatory biomarkers, especially those associated with neutrophilic inflammation. All participating patients gave written informed consent. The statistical analysis was performed with GraphPad Prism, version 10.1.1 (GraphPad Software, Boston, MA, USA). We compared FEV_1_ and pre- and post-ETI biomarkers using paired t-tests or Wilcoxon tests. Values of p < 0.05 were considered statistically significant.

A total of 8 pwCF (median age, 20.5 years) were enrolled. The mean interval between visits was 3 months. None of the patients had received any prior CFTR modulator therapy, and all were clinically stable during the visits. Demographic characteristics and bacterial colonization data are described in Table 1.

**Table 1:** Participant demographics, lung function, and respiratory culture results.

Patient	Demographics and clinical data				T0			T1		
	Sex	Age	Genotype (F508del status)	Previous P. aeruginosa colonization^a^	Previous B. cepacia complex colonization^a^	Z-score for FEV_1_	Respiratory sample culture^b^	Z-score for FEV_1_	Respiratory sample culture^b^	Time on ETI
		(years)								(months)
1	Male	34.20	Heterozygous	Free	Chronic	−5.7	*S. aureus* + *B. cepacia*	−5.61	*S. maltophilia + S. aureus*	0.7
2	Female	19.8	Homozygous	Free	Chronic	−3.27	MRSA	−2.31	MRSA	4.5
3	Male	19.5	Heterozygous	Free	Free	0.23	*S. aureus*	0.84	*S. aureus*	3.8
4	Male	21.1	Homozygous	Free	Free	−0.66	*S. aureus* + *B. cepacia*	0.62	*S. aureus*	2
5	Male	24.1	Heterozygous	Free	Chronic	−5.43	*B. cepacia*	−5.9	Negative	0.8
6	Male	21.7	Homozygous	Chronic	Free	−5.32	*P. aeruginosa*	−4.8	*P. aeruginosa*	9.1
7	Female	13.2	Homozygous	Free	Intermittent	−1.5	*B. cepacia* + *P. aeruginosa*	0.22	*S. aureus*	5.8
8	Female	7.4	Heterozygous	Chronic	Intermittent	N/A	*S. aureus*	N/A	Negative	2.3

*P. aeruginosa*: *Pseudomonas aeruginosa*; *B. cepacia*: *Burkholderia cepacia*; ETI: elexacaftor-tezacaftor-ivacaftor (therapy); *S. aureus*: *Staphylococcus aureus*; MRSA: methicillin-resistant *S. aureus*; and *S. maltophilia*: *Stenotrophomonas maltophilia*. ^a^Bacterial colonization status as defined per the Leeds criteria. ^b^Sputum or oropharyngeal swab culture.

**Figure 1:**
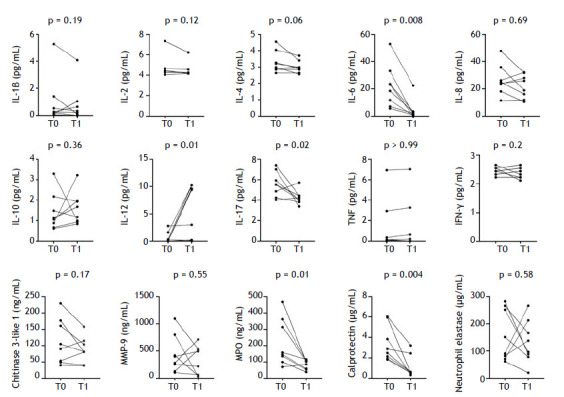
Inflammatory biomarkers at the T0 (pre-ETI) and T1 (post-ETI) time points. MMP-9: matrix metalloproteinase 9; MPO: myeloperoxidase; ETI: elexacaftor-tezacaftor-ivacaftor (therapy).

Seven patients underwent spirometry at both visits. The FEV_1_ showed significant improvement after treatment, with only one patient exhibiting a decline in FEV_1_ and only a mild decline at that (Table 1). All blood inflammatory biomarkers decreased at T1, except for IL-10 and IL-12. The difference between T0 and T1 was significant for IL-6, IL-12, IL-17, MPO, and calprotectin-20.8 vs. 1.5 (*p* = 0.008), 0.12 vs. 6.2 (p = 0.03), 5.52 vs. 4.14 (p = 0.04), 151.2 vs. 94.18 (p = 0.02), 2.67 vs. 0.59 (p = 0.005), respectively-as shown in Figure 1. 

Our findings are similar to those in the literature. Sheikh et al.^6^ demonstrated that blood levels of IL-6, IL-8, and IL-17A were significantly higher at baseline in pwCF than in healthy controls, and all three biomarkers showed a significant decrease after six months of ETI. In another study, Casey et al.^4^ showed a marked reduction in IL-6 after three months of ETI in 14 adults with advanced CF disease.^4^

The cytokine IL-17 has been of particular interest in the study of CF inflammation in recent years. This cytokine is secreted by various lymphocyte populations, particularly Th17 cells, in response to bacterial or nonbacterial stimuli, promoting neutrophil influx. In CF, there appears to be an imbalance between Th17 cells and Tregs, which normally counter-regulate them. In addition, there is a high prevalence of IL-17-producing neutrophils in peripheral circulation and sputum from pwCF.^7^ ^,^ ^8^ The increase in IL-12 observed after ETI use in our study sample is a novel finding; one hypothesis is that inflammation may have shifted from a Th17 pathway to a Th1 pathway, potentially indicating modulation of the adaptive immune response.

To our knowledge, this is the first study comparing calprotectin and MPO levels before and after ETI use. Calprotectin, a neutrophil-derived protein complex, is frequently elevated in the blood and sputum of pwCF. Levels have been found to increase during pulmonary exacerbations and decrease with appropriate antibiotic treatment, prompting studies to investigate this biomarker for potential disease monitoring.^9^ The neutrophil-derived enzyme MPO catalyzes the formation of potent oxidants, which participate in lung tissue damage in CF. The marked reduction of these biomarkers after treatment with ETI further corroborates the finding of decreased systemic neutrophilic inflammation.^10^

It is noteworthy that the one patient who did not show FEV_1_ improvement had a decrease in IL-6, IL-17, MPO, and calprotectin levels from T0 to T1. Other studies have also found no direct association between FEV_1_ improvement and the variation of the inflammatory marker levels in individual patients.^5^ One explanation may be that, in patients with advanced lung disease, the presence of structural abnormalities such as irreversible diffuse bronchiectasis has a more permanent impact on lung function despite a reduction in inflammation.

Our preliminary results corroborate prior initial findings that ETI therapy markedly reduces systemic inflammation. However, the precise mechanisms underlying this phenomenon are not yet completely understood. The main limitations of this study are its small sample size, heterogenous population, and varied follow up period. In addition, studying inflammatory biomarkers in blood is less specific to lung disease than is measuring them in sputum. However, these samples are easier to obtain in pwCF treated with ETI, who frequently show a marked reduction in sputum production. 

## References

[1] Bragonzi A, Horati H, Kerrigan L, Lorè NI, Scholte BJ, Weldon S (2018). Inflammation and host-pathogen interaction Cause and consequence in cystic fibrosis lung disease. J Cyst Fibros.

[2] Bruscia EM, Bonfield TL (2022). Update on Innate and Adaptive Immunity in Cystic Fibrosis. Clin Chest Med.

[3] Middleton PG, Mall MA, Drevínek P, Lands LC, McKone EF, Polineni D (2019). Elexacaftor-Tezacaftor-Ivacaftor for Cystic Fibrosis with a Single Phe508del Allele. N Engl J Med.

[4] Casey M, Gabillard-Lefort C, McElvaney OF, McElvaney OJ, Carroll T, Heeney RC (2023). Effect of elexacaftor/tezacaftor/ivacaftor on airway and systemic inflammation in cystic fibrosis. Thorax.

[5] Lepissier A, Bonnel AS, Wizla N, Weiss L, Mittaine M, Bessaci K (2023). Moving the Dial on Airway Inflammation in Response to Trikafta in Adolescents with Cystic Fibrosis. Am J Respir Crit Care Med.

[6] Sheikh S, Britt RD, Ryan-Wenger NA, Khan AQ, Lewis BW, Gushue C (2023). Impact of Elexacaftor-Tezacaftor-Ivacaftor on Bacterial Colonization and Inflammatory Responses in Cystic Fibrosis. Pediatr Pulmonol.

[7] Roesch EA, Nichols DP, Chmiel JF (2018). Inflammation in cystic fibrosis An update. Pediatr Pulmonol.

[8] Tan HL, Regamey N, Brown S, Bush A, Lloyd CM, Davies JC (2011). The Th17 Pathway in Cystic Fibrosis Lung Disease. Am J Respir Crit Care Med.

[9] Gray RD, Imrie M, Boyd AC, Porteous D, Innes JA, Greening AP (2010). Sputum and serum calprotectin are useful biomarkers during CF exacerbation. J Cyst Fibros.

[10] Jain R, Baines A, Khan U, Wagner BD, Sagel SD (2021). Evaluation of airway and circulating inflammatory biomarkers for cystic fibrosis drug development. J Cyst Fibros.

